# Epitheliocystis in Greater Amberjack: Evidence of a Novel Causative Agent, Pathology, Immune Response and Epidemiological Findings

**DOI:** 10.3390/microorganisms10030627

**Published:** 2022-03-15

**Authors:** Maria Chiara Cascarano, Maja Ruetten, Lloyd Vaughan, Maria Ioanna Tsertou, Dimitra Georgopoulou, Kleoniki Keklikoglou, Nikos Papandroulakis, Pantelis Katharios

**Affiliations:** 1 Department of Biology, University of Crete, 71003 Heraklion, Greece; mariachiaracascarano@gmail.com (M.C.C.); keklikoglou@hcmr.gr (K.K.); 2 Hellenic Centre for Marine Research (HCMR), Institute of Marine Biology, Biotechnology and Aquaculture (IMBBC), 71003 Heraklion, Greece; tsertou@hcmr.gr (M.I.T.); d.georgopoulou@hcmr.gr (D.G.); npap@hcmr.gr (N.P.); 3 Pathovet AG, 8317 Tagelswangen, Switzerland; maja.ruetten@pathovet.ch (M.R.); lloyd.vaughan@pathovet.ch (L.V.)

**Keywords:** *Seriola dumerili*, gills, intracellular bacteria, mucosal immunity, ionocytes, mucocytes

## Abstract

Epitheliocystis is a fish gill disease caused by a broad range of intracellular bacteria infecting freshwater and marine fish worldwide. Here we report the occurrence and progression of epitheliocystis in greater amberjack reared in Crete (Greece). The disease appears to be caused mainly by a novel Betaproteobacteria belonging to the *Candidatus* Ichthyocystis genus with a second agent genetically similar to *Ca.* Parilichlamydia carangidicola coinfecting the gills in some cases. After a first detection of the disease in 2017, we investigated epitheliocystis in the following year’s cohort of greater amberjack juveniles (cohort 2018) transferred from inland tanks to the same cage farm in the open sea where the first outbreak was detected. This cohort was monitored for over a year together with stocks of gilthead seabream and meagre co-farmed in the same area. Our observations showed that epitheliocystis could be detected in greater amberjack gills as early as a month following the transfer to sea cages, with ionocytes at the base of the gill lamellae being initially infected. Cyst formation appears to trigger a proliferative response, leading to the fusion of lamellae, impairment of gill functions and subsequently to mortality. Lesions are characterized by infiltration of immune cells, indicating activation of the innate immune response. At later stages of the outbreak, cysts were no longer found in ionocytes but were observed in mucocytes at the trailing edge of the filament. Whole cysts appeared finally to be expelled from infected mucocytes directly into the water, which might constitute a novel means of dispersion of the infectious agents. Molecular screening indicates that meagre is not affected by this disease and confirms the presence of previously described epitheliocystis agents, *Ca.* Ichthyocystis sparus, *Ca.* Ichthyocystis hellenicum and *Ca.* Similichlamydia spp., in gilthead seabream. Prevalence data show that the bacteria persist in both gilthead seabream and greater amberjack cohorts after first infection.

## 1. Introduction

Epitheliocystis is a common disease, mainly affecting the gills of a wide range of wild and farmed fish worldwide. A comprehensive review of the literature, largely predating molecular analysis, is covered by Nowak and LaPatra [1], with a more recent review addressing the insights forthcoming from molecular and genomic investigations by Blandford and colleagues [2]. Epitheliocystis is characterised by distinctive nodular intracellular inclusions (or cysts) containing the infectious agent residing and replicating within the host cell. Infection levels in this pathology can vary from low (also defined as subclinical, chronic, or non-pathological infections) to hyperinfections. Hyperinfections are mainly observed in farm environments, where high stocking densities and host stress are thought to facilitate the transmission and escalation of the pathology, although a study of epitheliocystis in wild and farmed brown trout (*Salmo trutta*) in a tributary of the Rhône in Switzerland found that the highest levels of infection occurred in wild trout populations, which the authors saw as reflecting the higher stress levels due to environmental fluctuations in the river system, as opposed to the more regulated conditions in fish farms [3].

During outbreaks, fish display respiratory distress, inappetence, lethargy and erratic swimming behaviour. Fish immune reactions to infection can include increased mucus production, inflammation, hyperplasia and proliferative cell response, causing, in severe cases, the destruction of lamellae and consequent disruption of oxygen uptake and other physiological functions of the gills. Mortalities are described mostly in younger fish, with rates usually low and progressive; nonetheless, events of sudden mass mortalities have also been reported, especially in fish larvae [4]. In specific areas with intense aquaculture activity, a year-to-year increase of mortality in juveniles due to epitheliocystis has been reported [5], raising concern over this pathology and stressing the need for a better understanding of the basic dynamics leading to mortality. It is still unclear why mortalities appear mostly in juveniles and what the other factors are that influence their rates. Different factors are believed to influence the occurrence of the disease, although generalisations are complicated by the huge diversity in bacteria causing the disease. For instance, transfer of Atlantic salmon from fresh water to sea water cages resulted in the loss of the epitheliocystis agent *Ca.* Clavochlamydia salmonicola from infected fish [6]. Changes or increases in water temperatures have often been cited, although these may merely reflect a coincidence of infectious periods with summer months (reviewed in [2]). In previously exposed older fish, immunity seems to be a crucial factor for the chronic manifestation of the disease (Katharios, personal observations).

Fish are the first jawed vertebrates to display a primordial adaptive immunity and are considered a model for comparative immunological studies in higher organisms [7]. Mucosal surfaces have been shown to possess their own mucosal-associated lymphoid tissue (MALT) [8] and therefore participate locally in the development of immunological memory. The gill milieu is continuously exposed to the external environment and represents a first barrier to the entrance of pathogens. Immune function in fish gill mucosa therefore includes both innate and adaptive components (gill-associated lymphoid tissue or GIALT) [9]. Contrary to other MALTS, the GIALT is additionally characterized by local aggregations of immune cells (mostly T but also B cells) resembling the structure of lymph nodes, named interbranchial lymphoid tissue or ILT [10,11].

Until now, the only epitheliocystis causative agent to have been cultivated, in vitro, is *Ca.* Syngnamydia salmonis, co-cultivated in the free living amoeba (FLA) and gill pathogen *Paramoeba perurans* [12]. Whether FLAs can be utilized for the cultivation of other epitheliocystis agents, such as the closely related *Ca.* Syngnamydia venezia [13], is not known. Several unsuccessful attempts have been made to recreate the ideal conditions of cultivation for these intracellular pathogens, with trials involving marine broths, marine agar and monolayers of EPC cell lines [5,14]. A key to developing such methods will surely come from developing a better understanding of the infectious process.

Epitheliocystis agents share the ability of infecting and replicating in the gills, but the infected cells seem to differ from study to study. Infected cell identification is in most cases uncertain because the intracellular replication of bacteria leads to cyst formation, distorting the normal appearance of the cell itself. As a result, many different types of cells have been identified as potential bacterial targets in epitheliocystis lesions. These include epithelial cells, ionocytes (also known as chloride or mitochondria rich cells), mucocytes and macrophages (reviewed in [1]). Although highly informative, the difficulty of interpreting these earlier studies is that the organisms described are now known not only to be different bacterial species but even to belong to different phyla. A “one-size-fits-all” solution for their cultivation is unrealistic.

The magnitude of the diversity of disease agents causing this disease is still largely unexplored. For many years, epitheliocystis was thought to be primarily caused by chlamydial pathogens [1,15,16,17,18], whereas, currently, a growing number of studies have identified a much broader range of phylogenetically unrelated and host-specific bacteria (reviewed in [2]) whose biology and mechanisms of action are still unclear. In Mediterranean aquaculture, epitheliocystis has been described up until recently mostly with histology and electron microscopy. This is the case for gilthead seabream (*Sparus aurata*) [15,19,20], European seabass (*Dicentrarchus labrax*) [21], common dentex (*Dentex dentex*) [22], sharpsnout seabream (*Diplodus puntazzo*) [4], red seabream (*Pagrus major*) [23,24], greater amberjack (*Seriola dumerili*) [25,26] and grey mullet (*Liza ramada*) [19,20]. Although these studies produced good quality imaging, they did not clarify the diversity of etiological agents in these hosts and, indeed, inspection of these images, through the extra-high-resolution lens of “hindsight”, reveals that many of the infections described were indeed mixed infections, involving quite different pathogens. In the last decade, molecular and genomic studies have started to fill this gap. Katharios and colleagues [27] identified a novel Gammaproteobacteria, *Ca.* Endozoicomonas cretensis, in epitheliocystis hyperinfections in sharpsnout seabream. Following this study, two novel bacteria (*Ca*. Ichthyocystis hellenicum and *Ca.* Ichthyocystis sparus) of a novel Betaproteobacterial genus (*Ca*. Ichthyocystis genus) were identified as being mainly responsible for the pathology in gilthead seabream, along with coinfections with the chlamydial agent *Ca*. Similichlamydia sp. [5,28].

Another substantial gap in the knowledge of this disease is the mode of transmission. Since epitheliocystis agents are characterised by an intracellular lifestyle, infected fish are likely their principal sources. The mode of transmission has never been investigated, which could be direct from fish to fish or may include intermediate hosts and/or environmental reservoirs. There are indications that epitheliocystis agents are host-specific, however; since it is a common practice in Mediterranean aquaculture to rear many different fish species in the same area, environmental interactions and host shifts should be taken into consideration.

Greater amberjack is a relatively new species in Mediterranean aquaculture. It is highly valued and highly appreciated by consumers because of its excellent taste. The species is sensitive to epitheliocystis and occasionally high mortalities have been reported in the literature [25].

In the present study, we report the occurrence of a novel epitheliocystis agent in greater amberjack reared at the experimental cage facilities of HCMR in Crete, Greece. To elucidate the dynamics leading to infection, a one-year monitoring survey of the fish reared in the farm was performed, including stocks of greater amberjack, gilthead seabream and meagre (*Argyrosomus regius*), following fish cohorts from the hatchery (inland facilities) prior to infection to the grow-out phase in sea cages. The aim of the study was to assess the occurrence and diversity of epitheliocystis agents over time in relation to parameters, such as fish size and temperature, that are thought to influence disease outbreaks and mortalities. Pathological findings were examined via histological, immunohistochemistry and micro-CT analysis. The results are extensively discussed to shape hypotheses that might explain the dynamics that lead to first infection, host response and outbreak resolution, along with the chronic persistence of pathogens in hosts and the means of transmission of the disease.

## 2. Material and Methods

### 2.1. Sampling Sites, Sample Collection and First Epitheliocystis Outbreak

Greater amberjack is currently reared at the research facilities of the Institute of Marine Biology Biotechnology and Aquaculture (IMMBC) of the Hellenic Center for Marine Research (HCMR) in Crete, Greece. The rearing takes place both at the AQUALABS, a land-based facility located in Gournes (Heraklion, Crete, Greece), and at SOUDA, a pilot-scale aquaculture cage farm located at Souda Bay (Chania, Crete, Greece). Groups of greater amberjack have been kept at SOUDA since 2004, when a group of hatchery-produced juvenile fish was transferred from the AQUALABS for reproduction and rearing-related research purposes. Since 2014, after establishing successful reproduction and larval rearing methods, new cohorts of fry/juveniles have been transferred to the pilot sea cages every year. Each cohort is kept in the sea for not less than one and a half to two years. Furthermore, different age classes of wild local populations of these fish are often observed swimming in the area near the cages.

A case of mortality of juvenile greater amberjacks reared at the pilot cage farm of HCMR in Souda bay (Crete, Greece) was reported in December 2017. Gill samples from diseased moribund fish (from now on referred to as cohort 2017) were obtained from a single cage and diagnosed with epitheliocystis (fresh mounts on gill filaments). Following diagnosis, sampling was repeated in the same cage twice more in the next 30 days (Supplementary File S1, Table S1). Collected samples included whole gill arches, single lamellae and manually micro-dissected cyst pools (obtained from a single fish). Samples were stored in 10% phosphate-buffered formalin (PBF), 96% ethanol and McDowell and Trump fixative (4F:1G), depending on the analysis to be performed (see following sections). Potential intermediate hosts for the epitheliocystis agents, including ascidian, bivalves, sponges and anemones, were sampled from cage nets in the fish farm during the second and third sampling. The samples were taken in duplicate. Additionally, unidentified biofouling organisms scraped from the nets and two-liter samples of seawater were also obtained during the outbreak. Samples were kept in 96% ethanol or frozen at −80 °C prior to DNA extraction and subsequently screened with epitheliocystis-related primers (see the section on *Pathogen identification*). Potential intermediate hosts were stored in PBF 10%.

### 2.2. Monitoring of Epitheliocystis in Souda Bay

In order to study the onset, progression, duration and possible resolution of the epitheliocystis infection in greater amberjack in Souda, a yearly monitoring plan of the three fish species farmed in the area (*Seriola dumerili*, *Argyrosomus regius* and *Sparus aurata*) was designed for the following year. From July 2018 to July 2019, eight samplings were performed to follow each fish cohort from the juvenile to the adult stage and from the inland hatchery to the netpen sea cages (Supplementary File S1, Table S2). At each sampling, fish size (weight and total length) and water temperature were recorded. For meagre and greater amberjack stocks, the first sampling was performed right before the transfer of the juveniles from the indoor tanks of the institute to the open sea cages (July 2018). These tanks are supplied with borehole sea water, virtually pathogen-free, and are therefore considered as a time zero prior to the infection. The following eight samplings were carried out at the sea cages in Souda bay. Gilthead seabream stocks were transferred to the cages in April 2018 and were first sampled two weeks after transfer (Supplementary File S1, Table S2). A total number of 92 gilthead seabream, 85 greater amberjack and 75 meagre were sampled during the monitoring period.

Sampling included partial gills arches that were preserved in 10% PBF, 96% ethanol and 4F:1G, depending on the analysis to be performed. If cysts were identified in the tissue (stereoscope examination) during the sampling procedure, additional gill samples were also taken in RNA*later* to allow future genomic study. In these cases, sampling was repeated within the next five days (sampling 09/2018 (2) in Supplementary File S1, Table S2).

Additional material from gill samples from the 2018 outbreak were stored in 96% ethanol and RNA*later* to perform whole-genome sequencing of the novel agent.

### 2.3. Pathogen Identification

DNA extraction from ethanol-stored gills (10–25 μg tissue), single cysts and a cysts pool was performed using a Qiagen DNeasy Blood and Tissue Kit (Purification of Total DNA from Animal Tissues protocol) with final DNA elution in 50 μL of RNase free water. Extracted DNA was PCR-screened for known epitheliocystis agents, such as *Ca.* Endozoicomonas spp., *Ca.* Ichthyocystis spp. and chlamydial spp., using genus-specific 16S rRNA gene primers and the PCR conditions specified in Table 1.

**Table 1:** Primers, primer sequences, and PCR conditions used in this study to amplify partial 16S rRNA of known epitheliocystis agents.

Agent	Primers	Sequence	Annealing (°C)	Extension Time	Product (bp)	Reference
Endozoicomonas spp.	Endo sp. F	5′-AGTAGGGAGGAAAGGTTGAAGG-3′	60	30”	400	[27]
	Endo sp. R	5′-CCCAGAATACAAGACTCCGGAC-3′				
Chlamydiales	16S IGF	5′-CGGCGTGGATGAGGCAT-3′	55	1.30′	1487	[17,29,30]
	16S B1	5′-TACGGYTACCTTGTTACGACTT-3′				
*Ca*. Ichthyocystis spp.	Ichthyo sp F	5′-AACTARGATGGTGGCGAGTG-3′	60	1′	900	[5]
	Ichthyo sp R	5′-CGCACATGTCAAGGGTAGG-3′				

Partial 16S rRNA gene amplicons were run on 1% agarose gel and visualized with ethidium bromide transillumination. Amplicons were named according to the fish species from which they had been isolated (ser, br and mg for greater amberjack, gilthead seabream and meagre, respectively), followed by fish number and sampling date (mmyy) and i or c to mark whether the amplicons were obtained with either *Ca.* Ichthyocystis or chlamydial specific primers (i.e., ser5-0119c is obtained from greater amberjack, fish number 5, sampled on January 2019, PCR-amplified with chlamydial primers). A selection of amplicons was purified with a QIAquick PCR Purification Kit (Qiagen), eluted in 50 μL of RNase free water and sent for Sanger dideoxy sequencing to CEMIA. Raw sequences were quality checked, assembled and extracted using FinchTV and Geneious Prime. The resultant sequences were deposited in NCBI under the accession numbers OL684917–OL684950.

To investigate the phylogeny of the causative agents, novel sequences were compared and blasted in the NCBI nucleotide database to find the closest match. Alignments were built using MUSCLE v3.8.31 [31] with default settings. Aligned sequences included BLAST matches, known close relatives and other known epitheliocystis agents. Maximum likelihood three (1000 bootstraps) was built using MegaX [32].

### 2.4. Potential Secondary Hosts and Environmental Screening

Molecular screening of potential intermediate hosts, net biofouling scrapes and water samplings were performed using the same DNA extraction kits and PCR primers as were used for the *Ca.* Ichthyocystis genus described in the section on pathogen identification. Filtering and digestive organs of filter-feeders, such as ascidians and bivalves, were dissected out and tested separately. For sponges, algae and biofouling organisms, whole tissue was homogenised prior to DNA extraction. Selected potential hosts growing on the nets included ascidians (pharynx and endostyle), *Mytilus* sp. bivalves (gills), *Pinctada* sp. bivalves (gills and digestive gland), anemones (whole body and tentacles) and sponges. Water samples were filtered with a sterile glass microfibre (4.7 diameter 0.02 μm pores) Whatman filter; DNA was extracted from the filter membrane and tested with the aforementioned PCR primers (Table 1).

### 2.5. Micro-Computed Tomography

One gill sample from a heavily infected fish fixed in 4F:1G was gradually dehydrated in 70% ethanol. Subsequently, the sample was stained overnight using 0.3% PTA dissolved in 70% ethanol [33]. A fish gill scan was performed with a SkyScan 1172 micro-tomograph (Bruker, Kontich, Belgium). The scanner uses a tungsten source and is equipped with an 11 PM CCD camera (4000 × 2672 pixels). The sample was scanned at a voltage of 60 kV and a current of 167 μA without a filter and a pixel size of 4.68 μm for a half rotation of 180°. Projection images were reconstructed into cross sections using SkyScan’s NRecon software (Bruker, Kontich, Belgium), which employs a modified Feldkamp’s back-projection algorithm. Volume renderings of the fish gills were created using SkyScan’s CTVox software (Bruker, Kontich, Belgium).

### 2.6. Histology and Immunohistochemistry

Samples fixed with PBF were progressively dehydrated in higher concentrations of ethanol (from 70–96% EtOH), embedded in glycol methacrylate resin (Technovit 7100, Heraeus Kulzer, Wehrheim, Germany) and cut in 4 μm sections with a microtome (RM 2245, Leica Biosystems, Nussloch, Germany). Sections were mounted on slides and stained with methylene blue/azure II/basic fuchsin (polychrome stain) [34] and with periodic acid–Schiff (PAS) using a commercial kit (Periodic Acid–Schiff (PAS) Stain Kit, Tcs Biosciences, Buckingham, UK).

Immunohistochemistry was performed at the PathoVet AG laboratory (Tagelswangen, Switzerland), after fixation in 4% buffered formalin. Samples were embedded in paraffin and cut sections were then mounted on positively charged slides and deparaffinized. Slides were subsequently stained with different antibodies. IBa-1 (Ionized calcium-binding adapter molecule 1) stain was performed with 25 min of antigen retrieval in Diva Decloaker (Biocare Medical, Pachecho, CA, USA) with antigen retrieval solution from Dako, pH 9 (Agilent Technologies, Glostrup, Denmark). The primary antibody, Anti IBa-1 rabbit (Fujifilm, Wako Chemicals Europe GmbH, Frauenfeld, Switzerland), was diluted 1:2000 and incubated for 60 min at room temperature. The secondary antibody, peroxidase DAB+ (Dako REAL Envision), was incubated for 30 min at room temperature.

Cytokeratin staining was performed using cytokeratin monoclonal antibody AE1/AE3 from Dako (Agilent Technologies, Glostrup, Denmark), proceeded by incubation for 30 min at room temperature with proteinase K (Agilent Technologies, Glostrup, Denmark). The primary antibody was diluted 1:400 and incubated for 60 min at room temperature. The secondary antibody, peroxidase DAB+ (Dako REAL Envision), was incubated for 30 min at room temperature.

Vimentin staining was performed with vimentin antibody (monoclonal mouse, clone V9) from Dako (Agilent technologies, Glostrup, Denmark), antigen retrieval of 25 min in Diva decloaker (Biocare Medical, Pachecho, CA, USA) with antigen retrieval solution from Dako pH 9 (Agilent Technologies Denmark). Primary antibody was diluted at 1:2000 and incubated for 30 min at room temperature. The secondary antibody, peroxidase DAB+ (Dako REAL Envision), was incubated for 30 min at room temperature.

### 2.7. Statistical Analysis

Husbandry data, including length, weight, growth rates and mortality of the reared fish stocks, as well as water temperature data, were recorded at the pilot farm as part of the regular rearing practices (Supplementary File S2, Data Sheets S4–S6).

Measurements of fish length and weight were obtained separately at each sampling point using the sampled fish (Supplementary File S1, Table S2).

Prevalence of infected fish was calculated as the percentage of fish found positive for epitheliocystis with either chlamydial or *Ca.* Ichthyocystis specific primers (Supplementary File S1, Table S2). It should be noted that a positive PCR signal was not always accompanied by observation of cysts on the tissue. Potential correlations between infection prevalence, temperature and fish size were investigated. To test whether temperature and fish size (length) can predict prevalence of infection, logistic models were fitted using the glm() function in R and best-fitted models were selected using AIC (Supplementary File S2, Data Sheets S2 and S3, code in Supplementary File S3). The results were plotted using GraphPad (Prism version 8.2.1).

Mortality of greater amberjack in 2017 and 2018 was calculated as the cumulative percentage of dead fish per cage. Mortalities were attributed to epitheliocystis by excluding other potential causes of fish deaths in the stock. Intensity of infection in greater amberjack samples was estimated by assessing cyst number per filament in the histological sections.

## 3. Results

### 3.1. Intensity, Prevalence and Mortalities

**Figure 1:**
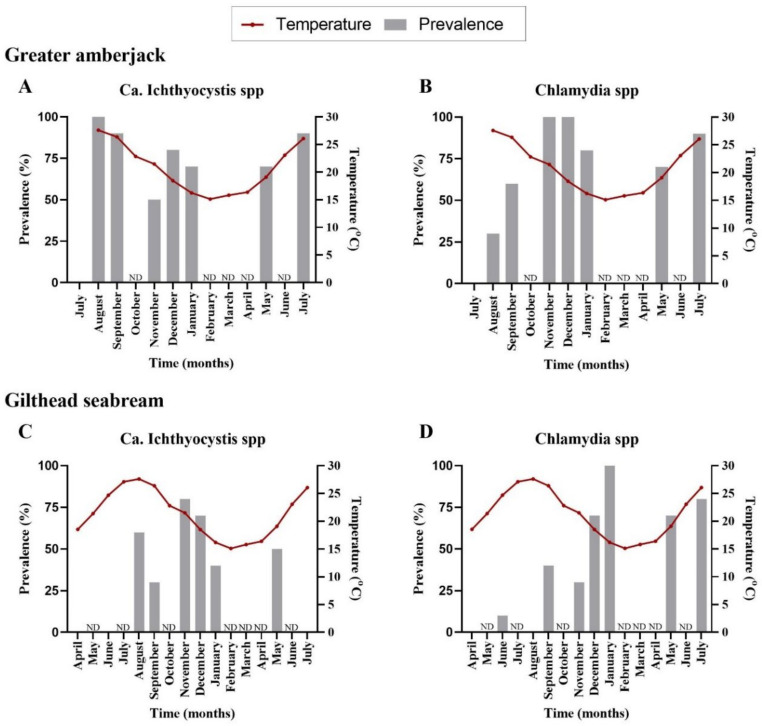
Prevalence of epitheliocystis agents in greater amberjack (**A**,**B**) and gilthead seabream (cohort 2018) (**C**,**D**). Water temperature is shown by the red line. ND (not determined) in the graphs marks months in which cohorts were not sampled.

The first detection of epitheliocystis in greater amberjack occurred one month after transfer with 100% prevalence for Ca. Ichthyocystis and 30% for chlamydia (Figure 1A,B, Supplementary Table S2). Mortalities attributed to the disease were observed immediately after first detection (two months after transfer, outbreak samples). Prevalence of infection was high during the following samplings, with more than 70% of the stock infected with both pathogens (Figure 1A,B, Supplementary Table S2). Infection intensity was quantified by cyst count in gills. Higher cyst numbers were found during the 2017 than the 2018 outbreak. In the 2017 cohort, outbreak samples in December (water temperature of 18.5 °C, approximate fish weight 200 g) displayed an approximate number of up 35 cysts per gill filament. In the 2018 cohort, outbreak samples for September (temperature 26.5 °C, approximate fish size 170 g) displayed an average of 4 cysts per gill filament and a maximum of 26 cysts per filament in heavily infected fish. In the following samplings fish found positive through PCR screening displayed an average of one cyst every two to eight filaments (sampling of November and December 2018, January and July 2019). One single fish in December 2018 showed a higher intensity of infection, with six cysts per filament.

We did not observe cysts in gilthead seabream histological sections; nevertheless, pathogens were detectable by PCR. Analysis of prevalence in infected gilthead seabreams indicated an initial signal for *Ca.* Ichthyocystis agents five months after transfer (6 fish out of 10) and for chlamydial pathogens three months after transfer (1 fish out of 10) (Figure 1C,D, Supplementary Table S2). The highest prevalence was recorded in November for *Ca.* Ichthyocystis sp. and January for chlamydia (Figure 1C,D, Supplementary Table S2).

In gilthead seabream and meagre stocks, no mortalities were attributed to epitheliocystis during the monitoring period. Meagre was, overall, not affected by epitheliocystis, except for one fish that was found positive for a chlamydial agent (see section on diversity of pathogens).

Neither the fish size (length) nor the temperature was correlated with the infection prevalence of the novel *Ca.* Ichthyocystis in greater amberjack (length estimate: −0.27, standard error: 0.16, statistic: −1.77, *p*-value: 0.08; temperature estimate: −0.03, standard error: 0.09, statistic: −0.36, *p*-value: 0.72). In contrast, both temperature and size (length) of the fish, were negatively correlated with prevalence of *Ca.* Ichthyocystis in gilthead seabream (temperature estimate: −0.18, standard error: 0.08, statistic: −2.30, *p*-value = 0.02; length estimate: −0.19, standard error: 0.08, statistic: −2.288, *p*-value: 0.02), showing that as the temperature and the length of the fish increased the presence of the pathogen (log-odds) decreased.

Regarding chlamydial agents, temperature was not correlated with the prevalence of these pathogens in greater amberjack (estimate: 0.02, standard error: 0.11, statistic: 0.15, *p*-value: 0.88), but fish length was (estimate: 0.24, standard error: 0.12, statistic: 2.02, *p*-value: 0.04). Chlamydial prevalence was correlated with both temperature and fish length in gilthead seabream (temperature estimate: −0.15, standard error: 0.08, statistic: −1.87, *p*-value: 0.06; length estimate: 0.23, standard error: 0.08, statistic: 2.92, *p*-value: 0.003).

**Figure 2:**
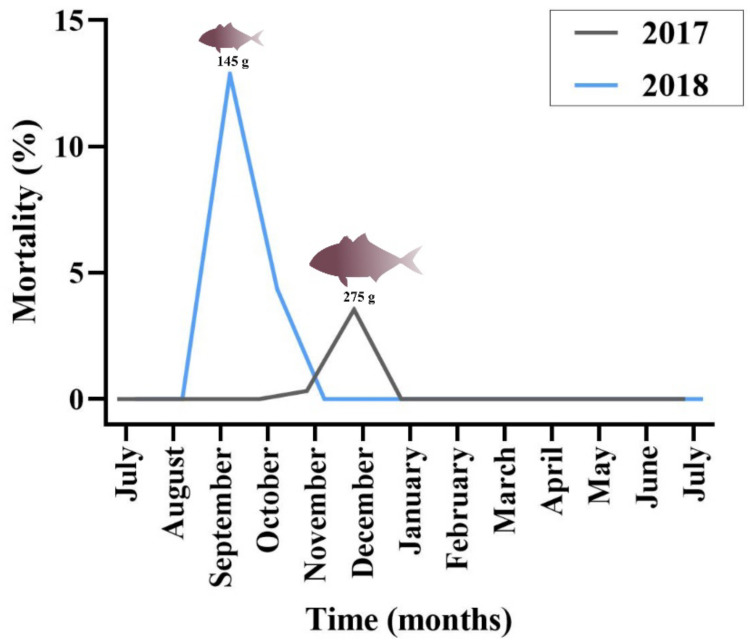
Mortality attributed to epitheliocystis in greater amberjack cohorts in 2017 and 2018. Fish weight during the outbreak is noted on the graph.

Regarding greater amberjack, cumulative mortality in the outbreak months was estimated at 4% in 2017 and 15% in 2018 (Figure 2). Cohort 2017 was transferred from the controlled environment (indoor tanks) to the sea cages in August, with an average fish weight of 2.3 g. Peak of mortality due to epitheliocystis in this stock was recorded 4 months after transfer, in December, with an average fish weight of 275 g. Cohort 2018 was transferred to cages in July (juveniles of 1.8 g) and peak of mortality was recorded in September with an average fish weight of 145 g. The outbreak in 2018 occurred two months after transfer and three months earlier than observed in the previous year.

In the 2018 greater amberjack cohort, the monogenean parasite *Zeuxapta seriolae* was occasionally found on fish gills. Infection levels were always low during the sampling dates. Increased parasitism was recorded in the farm in June 2019 (approximate fish weight 950 g) and mortalities up to 1.7% of the stock were attributed to this parasite in this month.

### 3.2. Diversity of Epitheliocystis-Causing Agents

**Figure 3:**
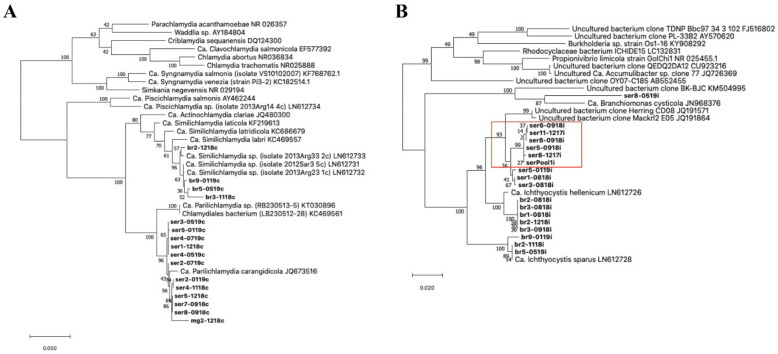
Phylogeny of the epithelyocystis agents encountered during this study. Phylogenetic analysis used alignments built with Muscle investigated with MEGA X using the maximum likelihood method and the Tamura–Nei model. Highest log likelihood trees for chlamydial lineage (**A**) and *Ca*. Ichthyocystis genus (**B**) are shown in scale. Numbers on branches indicate the percentage of trees in which the associated sequences clustered together and branch lengths represent the numbers of substitutions per site. The amplicons obtained in this study are shown in bold, with amplicon names given according to the fish species from which they were isolated (ser, br and mg, respectively, for greater amberjack, gilthead seabream and meagre) followed by fish number and sampling date (mmyy). (**A**) Chlamydial sequences obtained from meagre and greater amberjack clustered together with the previously described *Ca*. Parilichlamydia carangidicola isolated from the yellowtail kingfish (*Seriola lalandi*) in Australia [35]. Partial 16S rRNA sequences obtained from gilthead seabream clustered with *Ca*. Similichlamydia spp. previously described in gilthead seabream in Greece [28]. (**B**) Sequences obtained from gilthead seabream with *Ca*. Ichthyocystis sp. primers clustered with previously described *Ca*. Ichthyocystis sparus and hellenicum [5]. Sequences obtained from greater amberjack cluster with Clone Herring CD08 and Mackrl E05 and share the closest common ancestor with *Ca*. Ichthyocystis hellenicum. Amplicons retreived from outbreak samples of both 2017 and 2018 cluster together (red box). A single sequence shares a closest common anchestor with *Ca*. Branchiomonas cysticola, previously reported in epitheliocystis lesions in salmon [38].

Screening with chlamydial primers resulted in positive signals in a total of 97 out of 177 samples during the whole study, including 57 positive cases in greater amberjack (n = 57/85) and 40 in gilthead seabream (n = 40/92). Only one individual was positive for chlamydia in the stock of meagre (out of 75 sampled individuals). The single partial sequence retrieved from meagre (832 bp) had 98.47% similarity with a closest relative Ca. Parilichlamydia carangidicola (AN: JQ673516). From greater amberjack, we retrieved 11 sequences of around 1400 bp sharing up to 99.2% identity and clustering in two groups, one including five sequences with 0–4 bp variation and the other with six sequences with 0–9 bp variation (Figure 3A). These partial 16S rRNA sequences had a partial alignment (79% query cover), with up to 98.64% similarity with the partial 16S rRNA gene of *Ca.* Parilichlamydia carangidicola clone 25YTK11 (AN: JQ673516) from yellowtail kingfish (*Seriola lalandi*) in Australia [35]. Moreover, they shared up to 96.99% identity with two sequences retrieved from a *Ca.* Parilichlamydia sp. RB230513-5 (AN: KT030896) from epitheliocystis in cuckoo wrasse, *Labrus mixtus*, in Norway [36] and a Chlamydiales bacterium sp. LB230512-28 (AN: KC469561) from the ballan wrasse, *Labrus bergylta*, in Norway [37]. Finally, four partial sequences sharing above 97.7% pairwise identity were obtained with chlamydial primers in gilthead seabream. Three of these amplicons shared up to 98.56% similarity with *Ca.* Similichlamydia sp. isolate 2013Arg23_1c (AN: LN612732.1), while the fourth shared 98.2% identity with *Ca.* Similichlamydia sp. isolate 2013Arg33_2c (AN: LN612733.1) (Figure 3A); both *Ca.* Similichlamydia spp. were previously reported in gilthead seabream in other areas of Greece [28].

Screening with primers for the *Ca.* Ichthyocystis genus resulted in 59 positive samples in greater amberjack (n = 59/85 positive fish) and 33 positives in gilthead seabream (n = 33/92 positive fish). Moreover, cysts pooled from an epitheliocystis outbreak sample in greater amberjack (serPool1i) were positive for *Ca.* Ichthyocystis and negative for chlamydial primers. In gilthead seabream, we obtained eight partial 16S rRNA sequences (Figure 3B). Of these eight sequences, five (with lengths of up to 897 bp) showed up to 98.78% similarity with *Ca.* Ichthyocystis hellenicum (AN: LN612726.1) and three (with lengths up to 829 bp) had up to 99.76% similarity with Ca. Ichthyocystis sparus (AN: LN612728.1), previously described on the same host in other areas of Greece [5]. In greater amberjack, we obtained 10 partial 16S rRNA sequences using *Ca.* Ichthyocystis primers (Figure 3B). Nine of these sequences shared above 98.80% pairwise identity and were clustered in two different groups varying by 10–11 bp. One of these two clusters incorporated six close-to-identical sequences (99.9% pairwise identity), including all the amplicons obtained during both the 2017 and 2018 outbreaks (including the pooled cysts sample, serPool1i). The longest of these six partial sequences was selected as representative (ser5-0918i, length 895 bp) and used to classify the putative agent responsible for mortality in greater amberjack. This sequence had 96.10% identity with the recently described *Ca.* Ichthyocystis hellenicum (AN: LN906597.1) and 93.90% identity with *Ca.* Ichthyocystis sparus (AN: LN612728.1). BLAST results indicated an even higher percentage similarity in the alignment with two partial 16S sequences retrieved from NCBI (77% query cover); specifically, 97.68% identity with uncultured bacterium clone Herring_CD08 (AN: JQ191571.1) and 97.39% identity with clone Mackrl2_E05 (AN: JQ191864.1) obtained from the Pacific herring *Clupea pallasii* and the Pacific mackerel *Scomber japonicus*, respectively. Phylogenetic analysis showed that the nine sequences obtained from greater amberjack clustered within the genus Ca. Ichthyocystis and, considering that species distinction is defined between 94.5 and 98.7% nucleotide identity of the 16S rRNA gene [39], possibly belong to a novel species. Full-length 16S rRNA gene sequencing and whole-genome analysis, currently being carried out by our team, will clarify this further.

Out of the 10 sequences obtained with *Ca.* Ichthyocystis primers, 1, ser8-0519i, showed significant diversity. This 896 bp sequence shared 92.62% nucleotide identity with partial 16S rRNA sequences from *Ca.* Branchiomonas cysticola clones T200910 and LB2220920 (accession numbers JN807444.1 and OL314810.1, respectively) and 92.51% identity with the complete 16S ribosomal RNA gene of *Ca.* Branchiomonas cysticola from strain A1-483-L1 JN968376.1 retrieved from Atlantic salmon in Norway [38] (Figure 3B). This bacterium might represent a novel species and even belong to a novel genus clustering in proximity to the Branchiomonas genus.

Molecular screening of epitheliocystis on gills of the three stocks was always negative when fish were screened with *Endozoicomonas* primers.

Moreover, screening with epitheliocystis-specific PCR primers aiming to identify the pathogens in the environment surrounding the cages (including animals, biofouling and water samples), was in all cases negative.

### 3.3. Pathology in Greater Amberjack

#### 3.3.1. First Outbreak in Cohort 2017

A stock of greater amberjack juveniles (approximate weight of 200 g) in Souda bay (Crete, Greece) showed signs of distress, inappetence, change in swimming behaviour and low mortality rate (4%). Sampled fish displayed pale gills, an excess of mucus and sporadic infection with the polyopisthocotylean monogenean parasite *Zeuxapta seriolae* (one to two parasites per gill arch on 20% of fish). Fresh squash of gill filaments showed high intensity and prevalence of hyperplasic tissue with nodular focal lesions in interlamellar spaces.

**Figure 4:**
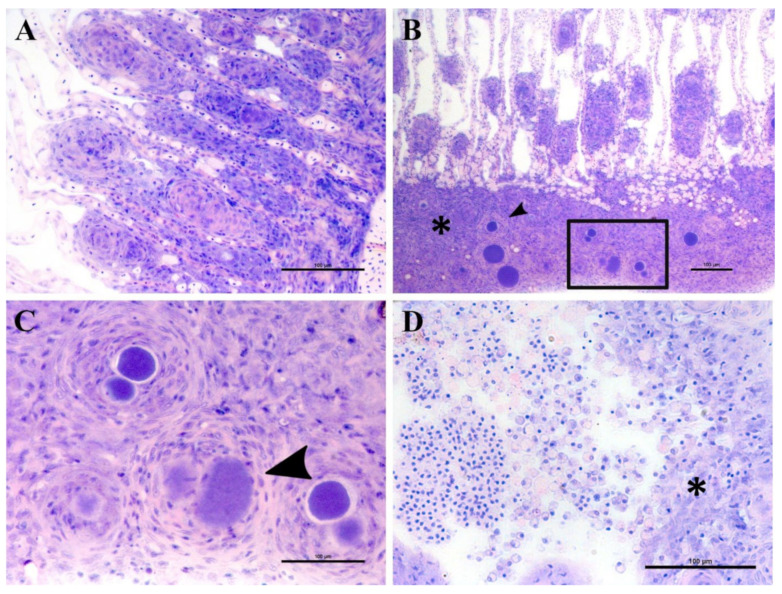
Histological sections of greater amberjack gills from the first outbreak of epitheliocystis (cohort 2017); polychrome stain. (**A**) Lamellar fusion (arrow) and extensive obliteration of the interlamellar spaces in a moribund fish. (**B**) Multifocal lamellar fusion (arrow) and extensive hyperplasic tissue (asterisk) including cysts (arrowhead). (**C**) Detail of hyperplastic area (frame) from (**B**), with densely packed cysts and ruptured cysts (arrowhead). (**D**) Macrophages infiltrating a hyperplasic area (asterisk).

Epitheliocystis was confirmed through histology with visible basophilically stained cysts (Figure 4B) containing finely granular inclusions (Figure 4C) surrounded by nodular focal layers of proliferative tissue. The extensive hyperplasic proliferative cell response was leading to fusion of lamellae and obliteration of several interlamellar spaces (Figure 4A,B). In moribund fish, lamellar destruction was more severe, with a higher percentage and a wider surface of fusion (Figure 4A). Proliferative tissue was expanding outside of the lamellar space in several points and hyperplasic focal lesions were also observed along the trailing edge of the filament (Figure 4B). Epitheliocystis (Figure 4C) and local infiltration of macrophages (Figure 4D) were observed in these areas.

#### 3.3.2. Monitoring of the 2018 Cohort

**Figure 5:**
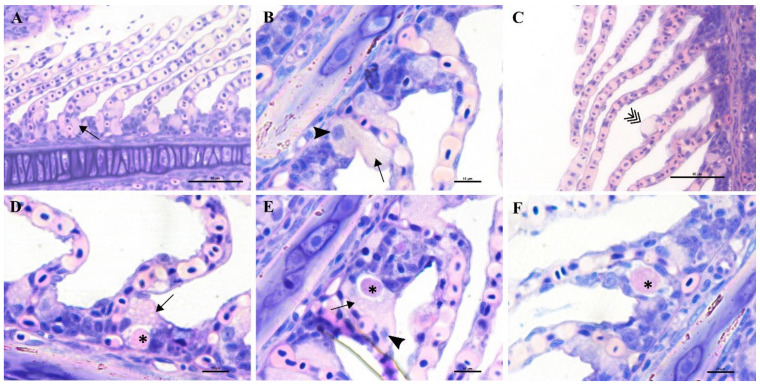
Histological sections of greater amberjack gills (cohort 2018) one month after transfer to sea cages (polychrome stain). (**A**,**B**) Normal gills showing ionocytes at the base of the lamellae (arrow); the basophilic nucleus of the ionocyte is shown by the arrowhead. (**C**) Enlarged cell along the lamella (multiple arrow). (**D**–**F**) Pictures obtained from samples positive for *Ca*. Ichthyocystis and negative for chlamydial agents. (**D**,**E**) Pinkish inclusions (asterisk) in a peripherical area of the cytoplasm of ionocytes at the base of the lamellae; the basophilic nucleus of the ionocyte is shown with the arrowhead. (**F**) Similar inclusion at the base of secondary lamellae (asterisk).

During the monitoring period, all sampled juvenile greater amberjack (cohort 2018) were positive for *Ca.* Ichthyocystis one month after their transfer to cages (August 2018, average weight 29 g). In this sampling, even though cysts were not found with gill histology, we nevertheless observed anomalies in several ionocytes at the base of the lamellae (Figure 5D–F). Some of these cells appeared enlarged and displayed an irregular, uneven, patchy cytoplasmatic content. In others (*Ca*. Ichthyocystis-positive/chlamydial-negative samples), it was possible to observe a dark pink rounded inclusion in a peripherical area of the cytoplasm (Figure 5D,E). These pinkish inclusions (10 μm diameter) were a common finding in the *Ca*. Ichthyocystis-positive samples and were mostly spotted at the base of the lamellae in contact with the filament, immediately above the epithelial layer of undifferentiated cells (Figure 5D–F). In rare cases we observed enlarged cells in other parts of the lamellae (Figure 5C).

**Figure 6:**
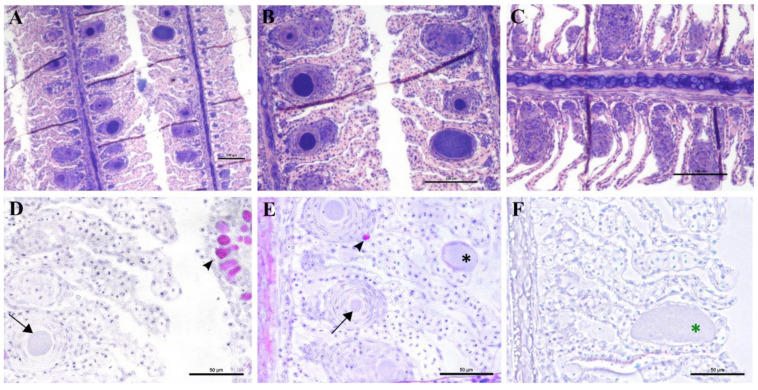
Histological sections of gills during and after the outbreak of epitheliocystis in greater amberjack (September 2018, approximate weight 170 g). (**A**–**C**) stained with polychrome stain; (**D**–**F**) stained with PAS stain. (**A**,**B**) Dark-stained cysts in the interlamellar spaces. Cysts are surrounded by layers of host proliferative tissue obliterating the interlamellar spaces. (**C**) Recovering fish with no cysts and residual proliferative response. (**D**,**E**) PAS-negative bacterial inclusions (arrow) and PAS-positive mucocytes (arrowheads). (**E**) Epitheliocystis inclusion in a PAS-positive cell (asterisk). (**F**) Epitheliocystis inclusion below the single layer of epithelial cells of the lamella (green asterisk).

An epitheliocystis outbreak in greater amberjack was recorded two months after the transfer of the fish to sea cages (approximate weight 170 g). During this month approximately 15% mortality was attributed to epitheliocystis since routine screening of the fish did not show other possible causes of death. Fish gills showed signs of inflammation and displayed multifocal lamellar fusion. Most cysts were observed at the base of the lamellae in contact with the filament (Figure 6A,B,D,E). These cysts were enclosed by varying degrees of proliferative response, consisting of focal layers of host proliferative tissue (Figure 6A,B,D,E). In most lesions, the cyst and surrounding proliferative tissue obliterated the interlamellar spaces from the filament to about half the height of the lamellae (Figure 6A,B), leaving only the apical portions of adjacent lamellae unaffected. We sporadically observed cysts in other positions of the lamellae (Figure 6E,F). These cysts were not delimited by any type of proliferative response and were located either above (Figure 6E) or below (Figure 6F) the single layer of epithelial cells of the lamellae. Cysts were always PAS-negative and mostly enclosed in PAS-negative cells (Figure 6D–F). Interestingly, cysts on the tips of the lamellae that did not display surrounding proliferative response were observed in PAS-positive cells (Figure 6E). The disease resolved spontaneously five days after the outbreak, with focal proliferative formations between lamellae (previously containing cysts) becoming gradually smaller (Figure 6C).

**Figure 7:**
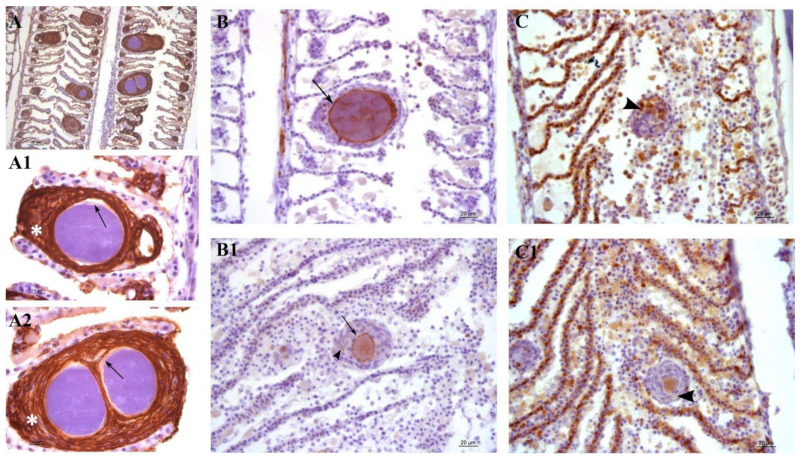
Immunohistology of greater amberjack gills during epitheliocystis outbreak (September 2018). Sections in panel (**A**) are stained with AE1/3 epithelial marker, those in panel (**B**) with vimentin stain and those in panel **C** with Iba-1 stain. (**A**,**A1**,**A2**) Multiple focal layers of proliferative epithelial tissue separate epitheliocystis inclusions from both filament and lamellae. (**A1**,**A2**) Proliferative tissue (asterisk) is made of cells that stain with epithelial markers. Between cysts and epithelial proliferative tissue, a thin rim (arrows) is observed that does not stain with epithelial markers. (**B**,**B1**) The rim between epitheliocystis and proliferative tissue is vimentin-positive (arrows). (**B1**) Some cells in between the focal layers of proliferative tissue display a vimentin-positive external membrane (arrowhead). (**C**,**C1**) Macrophages (arrowheads) infiltrate the proliferative tissue in several points.

Lesions in outbreak samples were examined with immunohistochemical markers. Epithelial marker staining showed that the focal layers of proliferating tissue surrounding the cysts were mainly composed of epithelial cells (Figure 7A,A1,A2). Iba-1 staining marked the infiltration of macrophages into the lesions (Figure 7C,C1). Between the multi-layered epithelial proliferative capsule and the bacterial inclusion, a thin rim was observed that did not stain with epithelial markers (Figure 7A1,A2). This area was vimentin-positive, indicating the presence of fibroblast-like cells or other vimentin-rich cells (Figure 7B,B1). Vimentin staining also marked the external membrane of some cells observed in the proliferating epithelial tissue surrounding the cysts (Figure 7B1).

**Figure 8:**
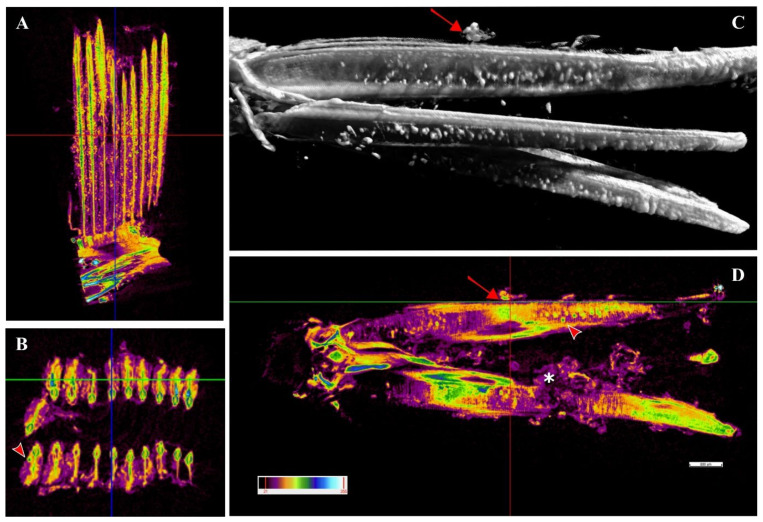
X-ray micro-computed tomography of greater amberjack infected gills (September 2018). (**A**,**B**,**D**) Two-dimensional cross sections of a partial gill arch seen along three different axes. Density of tissue is displayed with a range of colours from black to white. Denser structures are in white, blue and green. (**C**) Three-dimensional volume rendering of three filaments. Cysts are seen as yellow spherical structures between the secondary lamellae and when the section cuts through them they display a green (denser) colour (arrowheads). Proliferative epithelial tissue is also seen outside and between the filaments (asterisk) and, when density-inspected, the presence of cysts is revealed (arrows).

A single sample from a heavily infected fish was examined with micro-computed tomography (micro-CT). Cysts were observed along the whole length of the filament (Figure 8A,C,D) from the tip to the branchial arch (Figure 8A,C,D, Supplementary File S1, Figures S1 and S2) and moreover in the space between the hemibranch, where abundant proliferative tissue was observed (Figure 8C,D, Supplementary File S1, Figure S2). Cyst count was enormously facilitated by this analysis and revealed a much higher number of cysts than histology, with levels of infection reaching approximately 100 cysts per filament. Cyst size was measured, with diameters varying from 32–98 μm and an average of 47 μm. A file was produced (Supplementary File S4) including a video of the 3D volume rendering inspection of the sample.

**Figure 9:**
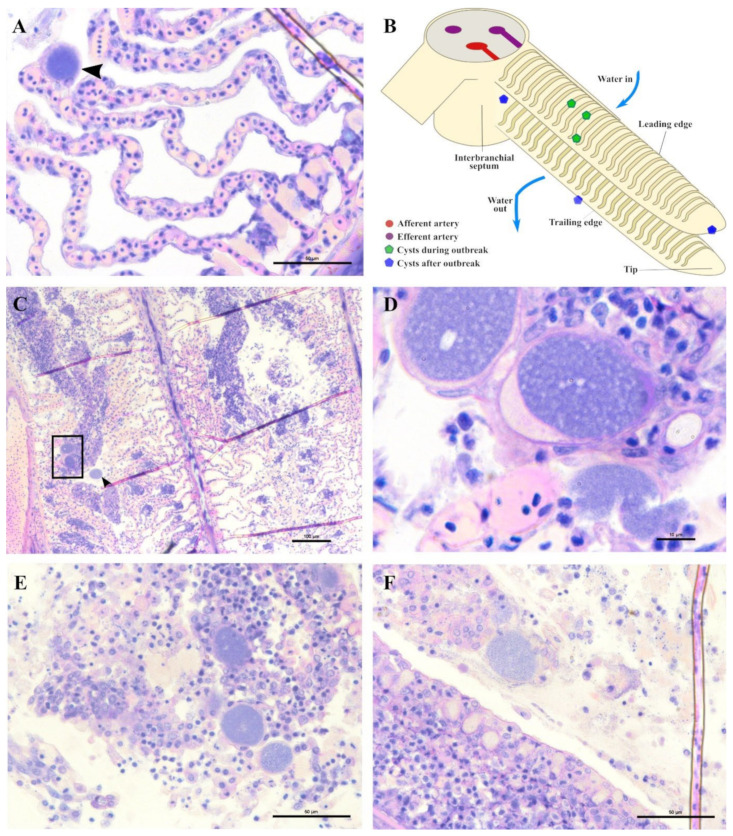
Monitoring of epitheliocystis in the months following the outbreak (cohort 2018). Polychrome stain of greater amberjack gills. (**A**) Cyst on the tip of the lamella, January 2019. (**B**) Illustration of a segment of the branchial arch showing the direction of the water flow and the different areas of the filament on which cysts were located during the outbreak (green stars) and after the outbreak (blue stars). (**C**) Cysts on the mucosa of the interbranchial septum, January 2019. (**D**) Higher magnification from the framed area of picture C. (**E**) Cysts on the tip of the filament, November 2018. (**F**) Cysts on the trailing edge of the filament, December 2018.

In the months following the outbreak, sampled fish were mostly still positive for the *Ca*. Ichthyocystis novel sp. and the chlamydial agent (see the section on the diversity of agents with prevalence data), but the lesions were always different from the ones previously described during the outbreak. The overall number of cysts was much lower, and it was not possible to identify any cysts in the interlamellar space at the base of the lamellae. We rarely observed cysts on the tip of the lamellae (Figure 9A) resembling the ones occasionally observed in the same position in the outbreak samples (Figure 6E). Most of the observed cysts were instead located on different areas of the epithelial surface of the filament (illustrated in Figure 9B). Specifically, cysts were spotted on the mucosa of the interbranchial septum (Figure 9C,D), on the epithelium of the tip of the filament (Figure 9E) and along the trailing edge of the filament (Figure 9D). Interestingly, none of these cysts were enclosed by hyperplasic layers of proliferative response.

**Figure 10:**
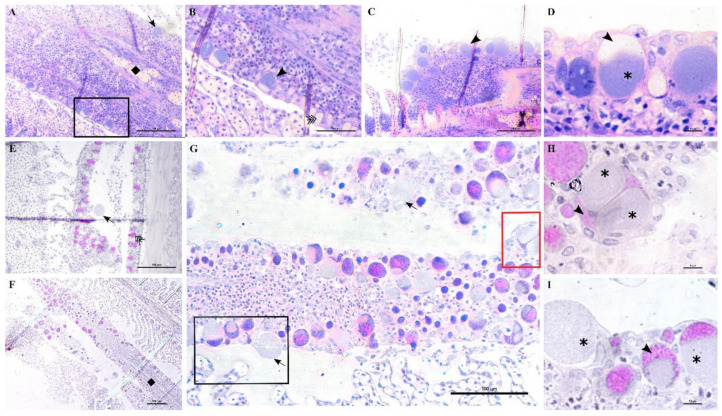
Anomalies in mucocytes in the trailing edge of filaments observed in the months following the epitheliocystis outbreak. Sections in (**A**–**D**) are stained with polychrome stain; sections in (**E**–**I**) are stained with PAS. (**A**,**B**) Sampling of December 2018; (**C**–**I**) sampling of January 2019. (**A**) Cyst (arrow) in the trailing edge of a filament; the section cuts along the afferent artery (rhombus). (**B**) Detail of anomalous mucocytes (arrowhead) showing blue granular content at the base of the cell; normal mucocytes display unstained cytoplasmatic content when stained with polychrome (multiple arrowheads). (**C**) Aggregate of anomalous mucocytes and cysts (arrowhead) at the base of the trailing edge of a filament. (**D**) Detail of the anomalous mucocytes. The cytoplasm of the cell is divided between an upper unstained portion (arrowhead) and a basal portion filled with blue granular content (asterisk). (**E**) Cysts (arrow) detached from the epithelium in proximity to the layer of mucocytes. Note that the cytoplasmatic content of normal mucocytes stains bright magenta (multiple arrowheads). (**F**) Trailing edges of both filaments of a hemibranch showing extensive aggregates of infected mucocytes. The section cuts along the afferent artery of one of the two filaments (rhombus). (**G**) Cysts (arrows) observed along the trailing edge of a filament in between anomalous mucocytes. (**H**) Higher magnification of the red-framed area in panel (**G**). Detail of a mucocyte containing two inclusions (asterisks) sharing cytoplasm filled with mucin (arrowhead). (**I**) Higher magnification from the black-framed area in panel (**G**). Different sizes of inclusions inside adjacent mucocytes. Mucus (arrowhead) is observed in the upper share of the cytoplasm, while the inclusion (asterisk) is positioned below. One large cyst is extruded from a ruptured cell.

In these samples, we observed anomalies in the peripherical layer of mucous cells on the trailing edge of the filament. Normal mucous cells are observed as pear-shaped cells (10 μm maximum width, up to 20 μm height) evenly and closely distributed at the outer layer of the epithelium (Figure 6D, Figure 10B,E) where they secrete their content. Mucin inside these cells is seen as unstained with polychrome stain (Figure 10B) and as bright magenta if stained with PAS (Figure 10E).

The anomalous mucocytes appeared slightly enlarged and, when stained in polychrome, showed a blue granular content in their basal portion (Figure 10A,B). This content seems to progressively increase from base to apex and finally occupy the whole internal volume of the cell (Figure 10D,E). Mucocytes then enlarge their diameter up to 50 μm (Figure 10G) and inclusions seem to be discharged from the epithelium (Figure 10G,I). Cysts are also observed detached from the epithelium in proximity to the layer of mucocytes (Figure 10E). Inside some of these mucocytes we could distinguish two bacterial inclusions sharing the same cytoplasm filled with mucin (Figure 10H).

Not all filaments showed anomalous mucus cells but, wherever one was observed, adjacent ones showed similar features (Figure 10C). Aggregations of infected mucus cells were mostly observed on the whole length of the trailing edge of the filament (Figure 10F,G) and on the trailing edge of the other filament in the same hemibranch (Figure 10F).

## 4. Discussion

### 4.1. Epitheliocystis-Causing Agents and Their Prevalence

The first description of epitheliocystis caused by members of the novel genus *Ca*. Ichthyocystis was in gilthead seabream in 2016 and 2017 [5,28]. In these studies, two novel species, *Ca*. Ichthyocystis hellenicum and *Ca*. Ichthyocystis sparus, were the principal causative agents for the pathology observed, while chlamydial pathogens, *Ca*. Similchlamydia species, had also co-infected the gills, contributing to the pathology to a lesser extent [5]. In the present study, we identified a novel uncultured epitheliocystis agent belonging to the *Ca*. Ichthyocystis genus in greater amberjack. Coinfecting chlamydial agents were also identified in this host with similarity to *Ca*. Parilichlamydia carangidicola, previously described in yellowtail kingfish in South Australia [35]. While the presence of the *Ca*. Ichthyocystis genus has not been reported or investigated in other fish species or other areas, our results, together with the NCBI sequences that suggest a possible presence of the genus in Pacific herring and Pacific mackerel, indicate that this novel class of pathogens might be distributed among a wider range of hosts. This hypothesis is consistent with the recent identification of a novel species of *Ca*. Ichthyocystis in the pompano *Trachinotus ovatus* (Cascarano and Katharios unpublished). These findings, taken together, should encourage the screening of other fish species for this genus and the clarification of the extent of diversity among these novel agents in different hosts.

Moreover, we have identified a novel Betaproteobacteria genus represented by a single partial amplicon obtained from greater amberjack gills in Crete the closest relative of which is *Ca*. Branchiomonas cysticola. Since the presence of this bacterium was detected only in a single fish and no data other than the partial sequence of the 16S rRNA is available, further investigation is required to support the novelty of this agent and its association with disease in fish.

We were able to identify different strains of the same agents previously described in gilthead seabream [5,28] from different regions of Greece, confirming that these bacteria are commonly found on this host in different areas of the Mediterranean Sea. Contrary to what was described earlier, we observed no inclusions nor an outbreak or epitheliocystis-related mortalities. It is important to note that other epitheliocystis agents belonging to the Betaproteobacteria can reside in fish gill cells without forming a clearly detectable cyst, as shown recently in Atlantic salmon [40]. Therefore, the lack of visible inclusions, together with the positive PCR results, might be associated not only with low bacterial loads but possibly with different modes of infection. While our results would be clarified by in situ hybridization and qPCR, we assume that the differences in disease intensity observed between this study and the previous ones on gilthead seabream [5,28] are most likely due to plain differences in local environmental parameters and stocking densities or to differences in local loads and/or the virulence of these strains.

The novelty of all the forementioned agents identified in this study must be confirmed by full-length 16S rRNA gene sequencing and/or whole-genome analysis. For the main *Ca*. Ichthyocystis agent associated with mortalities in greater amberjack, whole-genome sequencing has already been performed and genomic analysis is currently ongoing. Preliminary results indicate that it is indeed a new species of the genus (Cascarano et al., in preparation).

Our data on prevalence showed that gilthead seabream tested positive for the *Ca*. Ichthyocystis genus five months after transfer to the fish cages, while in greater amberjack a 100% prevalence was observed already one month after transfer and during the ensuing outbreak. This difference indicates a higher incidence rate in the population of greater amberjack, possibly due to a high local load of the bacteria (especially if they are indeed host-specific) and/or higher susceptibility to this *Ca*. Ichthyocystis strain. We observed a clear host-specificity in both chlamydial and *Ca*. Ichthyocystis agents for the co-farmed fish hosts we examined.

Interestingly, following initial detection, the prevalence of both chlamydial and *Ca*. Ichthyocystis pathogens was oscillating but persistent in the following samplings in both greater amberjack and gilthead seabream. This potentially indicates a long persistence of the infectious agents in the host (chronic infection) and/or a possibility of reinfection of the same fish in the future. An exception might be observed for the last sampling of July 2018 in gilthead seabream, where all sampled fish tested negative for the *Ca*. Ichthyocystis genus. Since this was observed only in the last sampling, it is impossible to know whether prevalence would increase again in the following months.

The results of the statistical analysis of prevalence suggest that an increase in temperature negatively affects the presence of both *Ca*. Ichthyocystis spp. and chlamydial agents in gilthead seabream, i.e., higher water temperature leads to lower prevalence. In greater amberjack we found no correlation between water temperature and presence/absence of epitheliocystis agents, indicating that other parameters might influence infection prevalence in this host.

The size of the fish (i.e., length) seems to be a significant predictor of the presence of both pathogens in both fish species studied. More specifically, the increase in size is negatively correlated with the presence of *Ca*. Ichthyocystis in both greater amberjack and gilthead seabream, while the opposite is the case for chlamydial agents (i.e., an increase in size leads to an increased probability of the presence of the pathogen in both species). It is important to consider that fish size increases over the time of the monitoring period. The size factor can therefore be translated into a time-in-cage factor, and, according to this, our prevalence results can imply that the longer the fish stays in the water, the higher the likelihood that the fish is infected with chlamydial agents, while, inversely, Ichthyocystis agents are most likely to infect hosts sooner after transfer.

### 4.2. Epitheliocystis in Greater Amberjack

The presence of visible lesions and the additional mortality data allowed us to build a more detailed picture regarding epitheliocystis onset and overall disease progression in greater amberjack. No specific immunostaining was performed in the current study to unambiguously label cysts and attribute them to either Ca. Ichthyocystis or Chlamydial agents. Even though such analysis would undoubtedly be helpful, we attempted to overcome this issue by differentiating between coinfected samples and samples infected with only one of the two pathogens. Particular attention was paid to the outbreak samples, these being the ones with the highest ratios of cysts per filament. Outbreak samples positive only for *Ca*. Ichthyocystis clearly displayed all the typical characteristics of the epitheliocystis outbreak, including interlamellar inclusions surrounded by proliferative tissue to different extents and fusion of adjacent lamellae. This subset of samples, interestingly, includes the single moribund fish of the 2017 outbreak. On the contrary, in chlamydia-only-positive samples (outbreak period), we could not detect cysts. Moreover, all 16S rRNA sequences obtained from micro-dissected single cysts from mixed infected outbreak samples were positive for the *Ca*. Ichthyocystis genus only. These observations collectively suggest that this novel agent is likely to be responsible for both the epitheliocystis outbreak and mortalities, while the chlamydial one might just be a minor coinfecting bacteria, similar to what has been shown for pathogens of the same genus in gilthead seabream [5].

In the positive samples of the fish one month after their transfer to sea cages, we observed peripherical cytoplasmatic inclusions in the ionocytes at the base of the lamellae. Since these inclusions were found in chlamydia-negative samples we assumed that these cells might represent a main first site of infection for the novel *Ca*. Ichthyocystis agent in greater amberjack. The position of these cells matches the sites of most inclusions during the following outbreak, where cysts and proliferating tissue progressively occupied the interlamellar space from the filament towards the apex of the lamellae. The optimal localisation of the inclusions along the filament during the outbreak was provided by micro-CT analysis. Differently from histology, in which visualisation of the sample is possible on one section at a time, the 3D visualisation from micro-CT rendering displays the whole sample at once. Cysts are readily identified due to the differential density that they display with respect to the rest of the tissue; their localisation along the whole tissue is unambiguous and their total quantification is enhanced.

Since we observed the first cytoplasmatic inclusions in early August and the disease was resolving by the middle of September, we can estimate the overall duration of the first infection to be about one month. Outbreak durations in other species of this bacterial genus have been estimated at around two weeks [5], but this might be related to the time it takes for cysts to fully form.

In 1990 and 1991 Crespo and colleagues and Grau and Crespo described epitheliocystis on farmed and wild amberjack cultivated in Port Andratx, Mallorca (Spain) using histology, scanning electron microscopy (SEM) and transmission electron microscopy (TEM) [25,26]. These authors described cysts surrounded by proliferative cell response in the trailing edge and in the interlamellar space of fish between 250 and 500 g. They first identified infecting organisms and “coccobacillary bodies” in ionocytes, specifying that in the latest stages of the pathology, once the cyst is fully formed, it is impossible to identify the nature of the infected cell. Histological pictures from Crespo and colleagues and the description of the lesions made by the authors [25] closely resemble our observations in the current study. No molecular data were produced by these authors, therefore we cannot conclusively demonstrate that the epitheliocystis agents are indeed the same. Still, we consider this to be a significant possibility, since epitheliocystis agents are mostly species-specific and both studies refer to the same host reared in the same area (Mediterranean Sea). These findings raise the question of whether strains of *Ca*. Ichthyocystis are also present in the Western Mediterranean, and, if so, what their relationship is to the strains found in Greece. Both greater amberjack and gilthead seabream are cultured in the Western Mediterranean; it would be fascinating to see whether the Eastern Mediterranean strains are present, and if so, whether the host-specificity is maintained. This information will be important to guide the development of treatment strategies, including vaccines.

### 4.3. Greater Amberjack Immune Response to First Infection

Following the infection of ionocytes, in more advanced stages of the infection we observed hyperplasia of the lamellar epithelium and proliferation of epithelial cells. Proliferation of undifferentiated epithelial cells in this area is commonly reported in fish exposed to heavy metals [41,42,43,44], drugs [45] and organic compounds [46,47], as well as during different kinds of gill infections [48,49], where it has been correlated with the downregulation of p53 tumour suppression protein and the overexpression of proliferating cell nuclear antigen PCNA [50]. This reaction was suggested to be a fish immune strategy aiming to increase the width of the thin tissue layer that separates the shallow respiratory capillaries from the harmful agent, preventing its permeation into the blood stream [44].

In the latest stages of the infection, we could observe breaking cysts and, in many points, concentric layers of proliferative tissue were the only pathological evidence remaining. We observed that in those fish that recovered from first infections, this proliferative epithelium undergoes degeneration, and the fish seem to return to a healthy state. A similar tendency has been shown in studies explaining interlamellar proliferation and gill remodelling in fish reared in polluted waters and subsequentially exposed to a clean environment [51]. Authors have suggested that proliferation is marked by a decrease in expression of PCNA and that an increase of apoptotic signals regulating cell death leads the final reshaping of the tissue.

In our case, proliferative tissue in the interlamellar space is shaped in nodular layers around the cyst, which is, moreover, interspersed with macrophages. This focal lesion appears to wall out the infected cell and resembles somewhat a granulomatous response. Since vimentin is considered a marker for the fibroblast lineage [52], we showed that a fibroblast-like vimentin-rich layer separates the cysts from the proliferative tissue. We cannot state whether this rim is made by fibroblasts or other kinds of cells that underwent degeneration with the progression of the disease, nor whether it includes the external membrane (and possible remnants) of the primary infected cell. Given that vimentin is being assigned an increasing number of physiological functions [53], the presence of those stained intermediate filament proteins in the lesions might be of greater importance and provide insights into immunological processes occurring in the lesion.

Vimentin is not commonly used to stain gill tissues, and, to our knowledge, such immunostaining has never been performed on epitheliocystis lesions, neither on healthy nor on diseased gills of greater amberjack or other carangidae. Most of what we know of the cellular functions of vimentin has been studied in other models (reviewed by [54]). Interestingly, this conserved intermediate filament has been defined as an antigenic target in autoimmunity and has also been associated with autoimmune disorders and proliferative lesions, such as sarcoidosis in humans, where it works as antigenic target for T and B cells [55,56]. It has been recognised as a species-conserved antigen receptor for natural killer cells in mammals and non-specific cytotoxic cells (NCCs) in fish [57]. Vimentin also has an antigenic function in tuberculosis and is overexpressed in macrophages infected by *Mycobacterium tuberculosis*, which are subsequentially targeted for killing [58]. Therefore, there is a possibility that the vimentin-rich ring surrounding the cyst in the described epitheliocystis lesion might act as antigenic target for other immune cells in epitheliocystis lesions.

Interestingly, we could identify some cells with vimentin-positive membranes infiltrating the proliferating tissue of epithelial cells. Vimentin has been shown to have different roles during bacterial infections [59], one of which is in transcellular migration and adhesion of lymphocytes [59]. This protein was shown to be secreted and surface-located in activated macrophages in response to pro-inflammatory signals (such as the cytokine tumour necrosis factor α, TNF-α) in a mechanism that is believed to promote pathogen trapping and killing [60]. Jaso-Friedmann and colleagues described NCCs in fish as bearing a vimentin-like surface domain [61]. These tissue-derived cells are part of the non-specific cellular immunity in fish and migrate in between cells to target infected cells for lysis [62]. Monteiro and colleagues described vimentin-positive ovoid cells in the interlamellar region of tilapia *Oreochromis niloticus* [63]. In the same approximate position, Koppang and colleagues stained MHC class II cells in Atlantic salmon (*Salmo salar*) [64]. The position of the vimentin positive cells in these two studies is of interest since they were located at the base of the lamellae, adjacent to mucous and mitochondria-rich cells, the same position in which we first observed the formation of the lesions. Whether the vimentin-positive cells interspersed in the lesion are activated macrophages or NCCs is yet to be clarified. The Iba-1 stain confirmed the presence of Iba-1-positive histiocytic cells in the same area. The presence of vimentin-positive cells and Iba-1-positive cells during the inflammatory process is a sign of activation of the immune response following first infection. Whether these cells migrate to primary infected cells and fuse to shape the vimentin-rich ring cannot be demonstrated in the present study. Future studies should attempt to clarify the eventual presence/position and function of vimentin-positive cells in healthy gills of greater amberjack.

### 4.4. Factors Influencing Mortality in Greater Amberjack

The extent of interlamellar space obliteration due to epithelial proliferation appears to be the primary cause of mortality, since in moribund fish the majority of lamellae were completely fused. When comparing mortality data from 2017 and 2018, we observed a higher mortality in fish of the 2018 cohort (mean weight 145 g) compared to those of the 2017 cohort (mean weight 275 g). Fish gill morphometrics gradually increase with fish age. Overall gill filament sizes increase during fish growth, increasing the overall respiratory surface as body mass increases [65]. If the relative amount of proliferative response caused by the infection of a single cell is constant but the height and length of the lamellae is bigger in older fish, we can expect a higher proportion of interlamellar space obliteration in younger fish. This means that, in older fish, the same level of proliferation might affect only a basal portion of the interlamellar space, leaving the apical part still functional and allowing the animal to resolve the infection without completely impairing the tissue respiratory function.

Mortality due to epitheliocystis in the study of Crespo and colleagues began in November, peaked in January–February and decreased in May, with a cumulative mortality of 85% of the stock [25]. This level of mortality, which is far above that reported here, and, of course, assuming the infective agent to be the same, might be explained by the different ranges of fish rearing temperatures. The authors stated that water temperature in Mallorca fluctuates from 13–25 °C and that the lowest temperatures were correlated with the highest mortality rates. In our study, we report an outbreak in 2018 at 26.5 °C and another in 2017 at 18.5 °C. Optimal rearing temperature for the greater amberjack is 26 °C and, moreover, it has been shown that if temperature decreases to 22 °C, there are no substantial effects on the growth of this fish [66]. On the contrary, a temperature of 17 °C has been set as the lower limit before the onset of deleterious effects for greater amberjack [66]. These optimal rearing temperature data agree with the growth rate that fish displayed in our study (data not shown), since below 22 °C the 2018 stock experienced a sudden drop in growth. We infer from this that a temperature of 13 °C is decisively suboptimal for rearing greater amberjack and might have been the cause of severe impairment of several physiological mechanisms (including immunity), explaining the severity of the disease reported in Mallorca.

We can conclude that both fish size and temperature seem to affect mortality rates. Comparing our mortality data from 2017 and 2018, though, we speculate that fish size has a much greater impact than temperature on mortality. In fact, when comparing the two outbreaks, we observed that in 2018 smaller fish in warmer temperatures experienced higher mortalities, while in 2017 bigger fish in lower temperatures exhibited lower mortalities.

Older fish co-farmed in the same area can represent a source of transmission of epitheliocystis agents, as we will discuss later in this manuscript. In gilthead seabream a year-by-year increase of mortality due to this disease was reported, and we could observe a similar trend when comparing the two outbreaks of 2017 and 2018 in greater amberjack. After the milder outbreak in 2017 (December), we experienced earlier and higher mortalities in 2018 (September). It is possible that the increased local load of bacteria persisting in the hosts after the outbreak in 2017 caused higher incident rates and earlier onset of the disease in 2018. Since, in each year, juveniles of the same size were transferred to cages in the same period, an earlier outbreak would have affected a smaller size class, causing higher mortalities. Considering that year class overlap is a common practice in fish farms, these results should be taken into serious consideration by stakeholders.

### 4.5. Chronic Stages of The Disease in Greater Amberjack

Most studies on epitheliocystis have investigated single outbreaks and no study, to our knowledge, has focused on the onset and progression of the disease in the same stock for an extended period. After the first detection of the pathogens, we reported oscillating prevalence during the year. Our prevalence data indicate that the bacteria persisted in the hosts for a prolonged time span after the outbreak and often went undetected, no mortality attributed to this disease being reported in adults.

In PCR-positive samples after the outbreak, it was not possible to observe any inclusion in the interlamellar area, where the first infection was clearly spotted. Moreover, no anomaly was found in ionocytes. The observed cysts were located in peripherical areas of the filament and, importantly, were never surrounded by any kind of proliferative response. Infected cells were stained PAS-positive, like mucocytes. Interestingly, we sporadically observed cysts in PAS-positive mucocytes in the upper part of the secondary lamellae also during the first outbreak, and those too were unaffected by epithelial proliferation.

Whatever the source of the first infection, it is unlikely that ionocytes would not be re-exposed to the same pathogen in time. Trained adapted immune response might protect these cells (or this area of the filament) from re-infection, while it is possible that the pathogen remains persistent and/or undetected inside the mucocytes.

It is important to note that the infected mucocytes were found in mucosal-like tissue in the interbranchial area, along the trailing edge and at the tip of the filament. These same areas have been characterized in other fish by the presence of the ILT [67] and should be therefore rich in T and B cells [10]. Dalum and colleagues redefined the interbranchial lymphoid tissue as divided into a proximal part (pILT) in the interbranchial septum and a distal part (dILT) extending along the trailing edges of the filament [68,69], the same areas in which we found the inclusions. Modifications of the normal structure of the ILT has been shown in gill disorders caused by amoebic gill disease (AGD) in Atlantic salmon (*Salmo salar*) [70]. Since this disease is also characterised by epithelial hyperplasia [71,72], it has been suggested that migration of immune cells from the lymphoid organ and epithelial proliferation triggered by the pathogen might be factors contributing to the reshaping of the tissue during infection [70]. Future study should characterize the ILT in healthy greater amberjack and investigate the reasons leading to what seems to be a lack of immune response following mucocyte infections in these areas. In mice and humans it has been shown that the same factor that promotes goblet cell differentiation and hyperplasia [73,74] is also associated with mucocyte metaplasia during airway epithelium infections, where it interferes with the innate immune response by binding TLR Toll-like receptor adapters and inhibits neutrophil recruitment [75,76].

Since no specific stain was performed to uniquely attribute these cysts to the novel *Ca*. Ichthyocystis agent, we cannot state with certainty that this bacterium can infect both ionocytes and mucocytes. While this analysis will, in the future, clarify the issue, we consider this to be a possibility since we excluded coinfection with chlamydial pathogens in these samples by PCR analysis. In case the novel pathogen can indeed infect both types of cells or transfer from one to the other, it would be interesting to characterise common properties of mucocytes and ionocytes in greater amberjack. Both cells are surely equipped with an extended internal tubular system of vesicles connected with the endoplasmic reticulum [77]. Moreover, it has been shown that ionocytes can excrete polysaccharidic substances that accumulate in their apical regions [78]. Interestingly, mucocytes have been recently attributed an additional immune function [79]. Sentinel mucocytes in mice can sample luminal material and secrete mucin in response, adapting mucus production to specific pathogens and inducing mucin secretion in adjacent mucocytes [80]. This mechanism has been observed in goblet cells in the apex of the epithelium (colon) and, interestingly, these same cells were subsequentially extruded from the epithelial layer [80].

### 4.6. Transmission of Pathogens Affecting Greater Amberjack and Pathogen Life Cycle Hypothesis

We observed cysts extruding outside of the tissue from mucocytes in the trailing edge of the filament. Considering that water flow in the gills is unidirectional, we can expect that whatever is released from the trailing edge can only flow outside the gills and cannot recirculate anywhere along the filaments. Extrusion of whole cysts from infected cells has never been described before and might represent a novel mechanism of dispersion for epitheliocystis agents. Future studies should include a consistent sampling of the water below and around the cages in both adult and juvenile cohorts in the months following outbreaks to confirm the presence of cysts and investigate the destiny of these pathogenic particles in the water column.

If floating cysts are a major means of fish-to-fish transmission of the bacteria, we can expect that they will not only enter the gills of a new host but will more likely be retained (filtered) rather than passing straight through, especially at the high flow rates expected for a fast-moving fish, such as greater amberjack. Moreover, in the scenario in which the agents can indeed infect both ionocytes and mucocytes and disperse as whole cysts out of the latter, the longer (and seemingly undetected) presence of these bacteria in mucocytes can indicate that these are preferential targets of infection, while first infection of ionocytes might just be accidental. The gill milieu is not a “static” environment (certainly not as static as that of internal organs) and water-circulating pathogens must overcome the high turbulence produced by the water flow. In this disturbed environment the outward surface of ionocytes and their microvilli “bulge” out of the epithelium and might represent an optimal point of attachment. This might potentially explain why we observe first infection only in ionocytes. Considering that first infection of ionocytes might lead to host mortality in juveniles, one might raise the question why a more specific targeting of the mucocytes has not been evolutionary selected as a unique host–pathogen mechanism of infection. A possible answer might be given by the fact that, if the pathogen is indeed water-circulating, the proliferative response triggered by the infection of ionocytes might increase the surface tissue between lamellae and facilitate the adhesion of other water-circulating bacteria, ensuring the success of the first infection. Moreover, if first infection activates a host response by promoting mucocyte proliferation (as suggested by the observed increased mucus production), it also facilitates subsequent stages of chronic infection in these cells.

Interestingly, Crespo observed the absence of microvilli on the apical pits of infected ionocytes in infected fish. The disruption of microvilli described by Crespo [25] could be a sign of the entrance of a pathogen in a ciliated cell, a mechanism described as attaching and effacing (A/E) in enteropathogenic bacteria [81,82,83]. In Gram-negative bacteria, such as *Neisseria meningitidis*, the first adhesion in A/E is mediated by type IV pili [84] and has been recognised to be tissue/cell specific [85]. In this regard, other bacteria of the Ca. Ichthyocystis genus have been sequenced and their genomes described [86]. Between the different virulence attributes of these bacteria, the presence of type IV pili has been discussed.

Future studies including transmission electron microscopy will investigate the nature of the infected cells during the different stages of infection and will help to identify the morphology of bacteria in the cysts observed in both ionocytes and mucocytes. These studies will hopefully explain some of the findings and confirm or disprove the hypothesis discussed here.

Further genomic analysis of the novel pathogen described in greater amberjack in Greece will shed light on virulence attributes of this novel bacteria and help to shape theories about the mechanism of the host-selective first attachment in the gills. The additional tissue stored from the outbreak of 2018 is currently being analysed and pooled cysts have been subjected to whole-genome sequencing. Genomic sequencing is going to provide infection clues additional to those included in the present work and possibly explain some of the pathological findings described herein.

## 5. Conclusions

Fish are already considered to be a model for comparative immunological studies. Intracellular infection by epitheliocystis agents challenges both innate and acquired immunity in the same localized area. If the limitation caused by the current difficulties in establishing in vitro models is bypassed, the study of this disease in the future will provide insights into basic immunity mechanisms and possibly expose novel functions of cells in the mucosa.

The *Ca*. Ichthyocystis genus includes a growing number of species affecting the gills of fish in the Mediterranean Sea. A novel species was identified in this study in greater amberjack reared in Greece whose pathological features resemble those described by Crespo and colleagues in 1990 in Spain. The 16S rRNA sequence of the novel species clusters in proximity to two BLAST-deposited clone sequences obtained from Pacific herring and Pacific mackerel, indicating the possibility of at least five different intracellular bacteria species causing epitheliocystis in four different Mediterranean fish hosts.

This disease in greater amberjack involves two different types of target cells, one dominant during a first acute infection phase and the other dominant during the chronic stage of the disease. Whole-cyst extrusion from infected mucocytes and shedding directly into the water is a possible new mode of dispersion of epitheliocystis agents. Chronic infection in adults might contribute to infection in younger fish if stocks of different age class are co-farmed. Fish year class overlap can contribute to an increase in the local environmental load of these pathogens in a single area and therefore lead to an increase of year-to-year mortalities. Mortalities seem to be related to the degree of obliteration of the interlamellar space caused by the proliferative response and might be exacerbated in younger fish with smaller gill morphometrics.

The insights into both host response and pathogen transmission provided by the present study propose alternative theories for future research towards the increase of knowledge of this poorly understood, emerging disease.

## Data Availability

All data included in this study are provided in the Supplementary Materials. Genomic sequences have been uploaded to GenBank.

## Supplementary Materials

The following supporting information can be downloaded at: https://www.mdpi.com/article/10.3390/microorganisms10030627/s1. Supplementary Material Data S1; Supplementary Material Data S2; Supplementary Material Data S3: Code for statistical analysis; Video S1: 3D Micro-CT video.
