# Supplementary material for: Epitheliocystis in Greater Amberjack: Evidence of a Novel Causative Agent, Pathology, Immune Response and Epidemiological Findings
Source: Microorganisms. 2022 Mar 15;10(3):627. doi: 10.3390/microorganisms10030627 (PMC8949381; doi:10.3390/microorganisms10030627)
Supplement: Supplementary file 1 [file microorganisms-10-00627-s001.zip › Suplementary File S4.pdf]

Link to supplementary file 4: video from micro-CT
