# Supplementary material for: Epitheliocystis in Greater Amberjack: Evidence of a Novel Causative Agent, Pathology, Immune Response and Epidemiological Findings
Source: Microorganisms. 2022 Mar 15;10(3):627. doi: 10.3390/microorganisms10030627 (PMC8949381; doi:10.3390/microorganisms10030627)
Supplement: Supplementary file 1 [file microorganisms-10-00627-s001.zip › Supplementary_File_S1.pdf]

SUPPLEMENTARY MATERIAL DATA

Supplementary Table S1. Samplings data from the first outbreak of epitheliocystis in Souda bay (Crete, Greece) in Greater amberjack (cohort 2017).

Date (dd/mm/yyyy)	Temp °C	number of fish sampled	Approximate fish weight (g)
08/12/2017	18	5	-
11/12/2017	18	11	200 g
10/01/2018	16.5	6	300 g

Supplementary Table S2. Sampling data (cohorts 2018) from the monitoring program of epitheliocystis in Souda bay (Crete, Greece). When location is HCMR, sampling was performed in indoor tanks right before the transfer to the sea cages. In bolt outbreak samples. Sampling 09/2018 (2) was a duplicate sampling to evaluate the progression of the outbreak.

			**gilthead seabream**				**greater amberjack**				**meagre**			
Date (mm/yyyy)	location	Temperature (°C)	Sample size	Mean length (cm)	Mean weight (g)	Prevalence Chlam/ Ichthyo	Sample size	Mean length (cm)	Mean weight (g)	Prevalence Chlam/ Ichthyo	Sample size	Mean length (cm)	Mean weight (g)	Prevalence Chlam/ Ichthyo
04/2018	Souda	18.5	10	7	5.5	0/0	-	-	-	-	-	-	-	-
06/2018	Souda	24.7	12	11	24	1/0	-	-	-	-	-	-	-	-
07/2018	HCMR	-	-	-		-	10	11	1.8	0/0	5	5	2	0/0
08/2018	Souda	27.6	10	16	69	0/6	10	13	29	3/10	10	10	14	0/0
09/2018	Souda	26.4	10	17	86	4/3	**10**	**23**	**170**	**6/9**	10	16	62	0/0
09/2018(2) *	Souda	-	-	-	-	-	5*	22	149	4/4	-	-	-	-
11/2018	Souda	21.5	10	20	152	3/8	10	31	459	10/5	10	27	233	0/0
12/2018	Souda	18.5	10	22	230	7/7	10	32	496	10/8	10	28	274	1/0
01/2019	Souda	16.2	10	24	246	10/4	10	35	578	8/7	10	26	248	0/0
05/2019	Souda	19.1	10	26	330	7/5	10	38	812	7/7	10	33	361	0/0
07/2019	Souda	26	10	28	376	8/0	10	26	1252	9/9	10	29	300	0/0

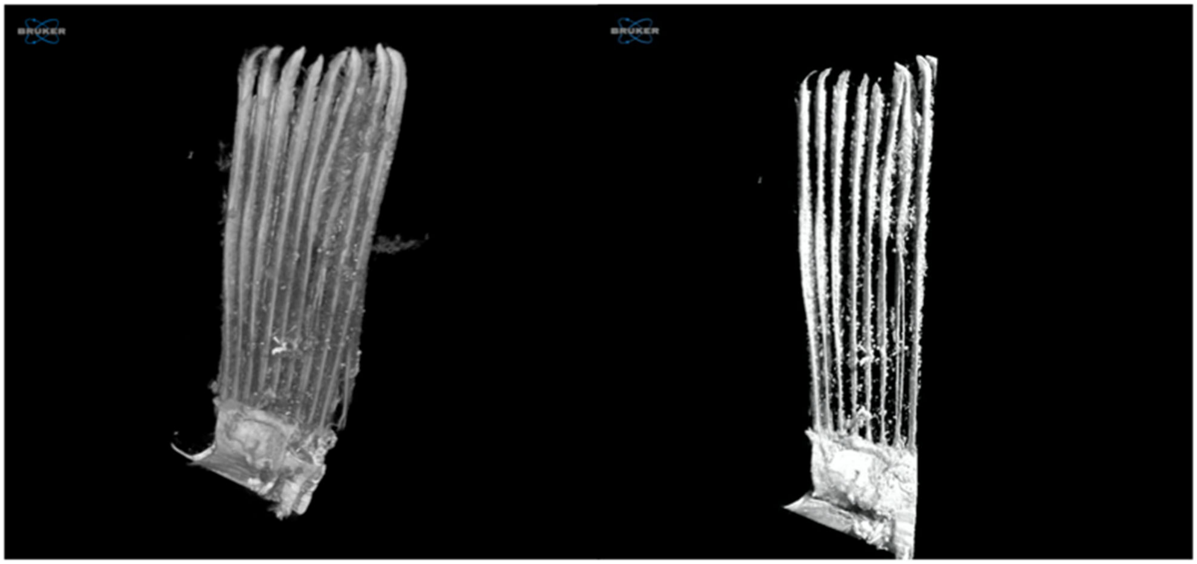

Supplementary Figure S1. 3D rendering of epitheliocystis infected gill fragment from Greater amberjack. In the second image denser structure are enhanced, showing presence of cysts in all the lengths of the filaments.

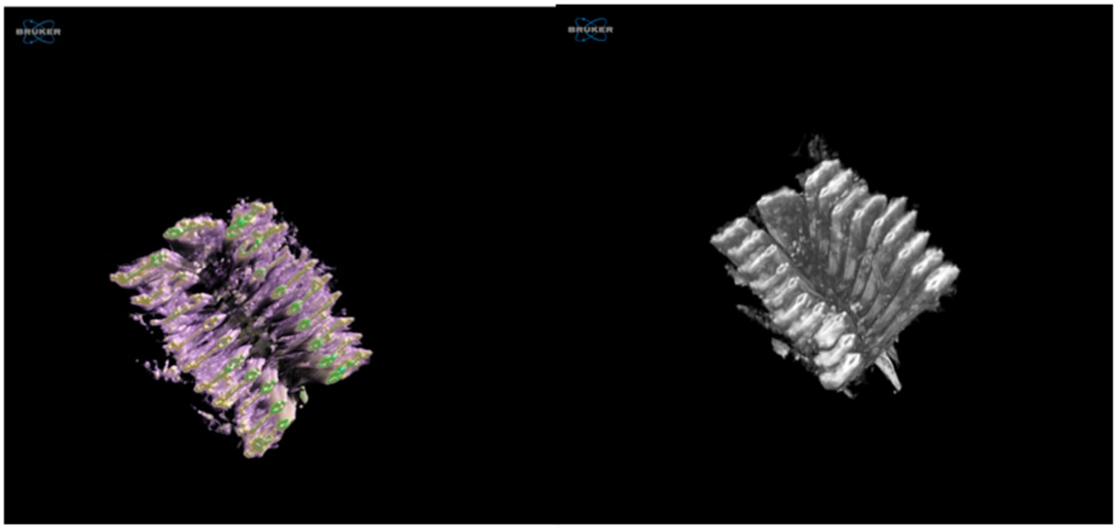

Supplementary Figure S2. 3D rendering of epitheliocystis infected gill fragment from Greater amberjack. The section shows the area between the holobranchs in proximity to the gill arch. The area between the hemibranchs is interspersed by proliferative tissue.
