# Supplementary material for: Epitheliocystis in Greater Amberjack: Evidence of a Novel Causative Agent, Pathology, Immune Response and Epidemiological Findings
Source: Microorganisms. 2022 Mar 15;10(3):627. doi: 10.3390/microorganisms10030627 (PMC8949381; doi:10.3390/microorganisms10030627)
Supplement: Supplementary file 1 [file microorganisms-10-00627-s001.zip › Supplementary_File_S3.pdf]

R CODE

```
############## LOGISTIC FINAL #################
############## 1. SERIOLA     #################
# i. Ca. Ichthyocystis

# TEMPERATURE
mylogit <- glm( presence ~ temp  , data = logisticData, family = "binomial")

summary(mylogit)
confint(mylogit)
plot(mylogit)
exp(coef(mylogit))

probabilities <- predict(mylogit, type = "response")
predicted.classes <- ifelse(probabilities > 0.5, "pos", "neg")
head(predicted.classes)
logit = log(probabilities/(1-probabilities))


# WEIGHT
mylogit <- glm( presence ~ weight  , data = logisticData, family = "binomial")

summary(mylogit)
confint(mylogit)
plot(mylogit)
exp(coef(mylogit))

probabilities <- predict(mylogit, type = "response")
predicted.classes <- ifelse(probabilities > 0.5, "pos", "neg")
head(predicted.classes)
logit = log(probabilities/(1-probabilities))

# LENGTH
mylogit <- glm( presence ~ length  , data = logisticData, family = "binomial")

summary(mylogit)
confint(mylogit)
plot(mylogit)
exp(coef(mylogit))

probabilities <- predict(mylogit, type = "response")
predicted.classes <- ifelse(probabilities > 0.5, "pos", "neg")
head(predicted.classes)
logit = log(probabilities/(1-probabilities))


# MULTI-VARIABLE

mylogit <- glm( presence ~ temp + length + weight , data = logisticData, family =
"binomial")
```

```
summary(mylogit)
confint(mylogit)
plot(mylogit)
exp(coef(mylogit))

probabilities <- predict(mylogit, type = "response")
predicted.classes <- ifelse(probabilities > 0.5, "pos", "neg")
head(predicted.classes)
logit = log(probabilities/(1-probabilities))


# # # # # # # # # # # # # # # # # # # # # # # # # # # # # # # # # # # # #
# ii. Chlamydia

# TEMPERATURE
mylogit <- glm( presenceChlam ~ temp  , data = logisticData, family = "binomial")

summary(mylogit)
confint(mylogit)
plot(mylogit)
exp(coef(mylogit))

probabilities <- predict(mylogit, type = "response")
predicted.classes <- ifelse(probabilities > 0.5, "pos", "neg")
head(predicted.classes)
logit = log(probabilities/(1-probabilities))


# WEIGHT
mylogit <- glm( presenceChlam ~ weight  , data = logisticData, family = "binomial")

summary(mylogit)
confint(mylogit)
plot(mylogit)
exp(coef(mylogit))

probabilities <- predict(mylogit, type = "response")
predicted.classes <- ifelse(probabilities > 0.5, "pos", "neg")
head(predicted.classes)
logit = log(probabilities/(1-probabilities))

# LENGTH
mylogit <- glm( presenceChlam ~ length  , data = logisticData, family = "binomial")

summary(mylogit)
confint(mylogit)
plot(mylogit)
exp(coef(mylogit))
```

```
probabilities <- predict(mylogit, type = "response")
predicted.classes <- ifelse(probabilities > 0.5, "pos", "neg")
head(predicted.classes)
logit = log(probabilities/(1-probabilities))


# MULTI-VARIABLE

mylogit <- glm( presenceChlam ~ temp + length + weight, data = logisticData, family =
"binomial")

summary(mylogit)
confint(mylogit)
plot(mylogit)
exp(coef(mylogit))

probabilities <- predict(mylogit, type = "response")
predicted.classes <- ifelse(probabilities > 0.5, "pos", "neg")
head(predicted.classes)
logit = log(probabilities/(1-probabilities))


############## 2. SEABREAM     #################
# i. Ca. Ichthyocystis

# Temperature
mylogit <- glm( presence ~ temp  , data = logisticDataSeabream, family = "binomial")
summary(mylogit)
confint(mylogit)
plot(mylogit)
exp(coef(mylogit))


probabilities <- predict(mylogit, type = "response")
predicted.classes <- ifelse(probabilities > 0.5, "pos", "neg")
head(predicted.classes)
logit = log(probabilities/(1-probabilities))

# Weight
mylogit <- glm( presence ~ weight  , data = logisticDataSeabream, family = "binomial")
summary(mylogit)
confint(mylogit)
plot(mylogit)
exp(coef(mylogit))


probabilities <- predict(mylogit, type = "response")
predicted.classes <- ifelse(probabilities > 0.5, "pos", "neg")
head(predicted.classes)
logit = log(probabilities/(1-probabilities))
```

```
# Length
mylogit <- glm( presence ~ length  , data = logisticDataSeabream, family = "binomial")
summary(mylogit)
confint(mylogit)
plot(mylogit)
exp(coef(mylogit))


probabilities <- predict(mylogit, type = "response")
predicted.classes <- ifelse(probabilities > 0.5, "pos", "neg")
head(predicted.classes)
logit = log(probabilities/(1-probabilities))

# Multi-variable
mylogit <- glm( presence ~ temp + weight  , data = logisticDataSeabream, family =
"binomial")
summary(mylogit)
confint(mylogit)
plot(mylogit)
exp(coef(mylogit))


probabilities <- predict(mylogit, type = "response")
predicted.classes <- ifelse(probabilities > 0.5, "pos", "neg")
head(predicted.classes)
logit = log(probabilities/(1-probabilities))


# # # # # # # # # # # # # # # # # # # # # # # # # # # # # # # # # # # # #
# ii. Chlamydia


# Temperature
mylogit <- glm( presenceChlam ~ temp  , data = logisticDataSeabream, family = "binomial")
summary(mylogit)
confint(mylogit)
plot(mylogit)
exp(coef(mylogit))


probabilities <- predict(mylogit, type = "response")
predicted.classes <- ifelse(probabilities > 0.5, "pos", "neg")
head(predicted.classes)
logit = log(probabilities/(1-probabilities))

# Weight
mylogit <- glm( presenceChlam ~ weight  , data = logisticDataSeabream, family =
"binomial")
summary(mylogit)
```

```
confint(mylogit)
plot(mylogit)
exp(coef(mylogit))


probabilities <- predict(mylogit, type = "response")
predicted.classes <- ifelse(probabilities > 0.5, "pos", "neg")
head(predicted.classes)
logit = log(probabilities/(1-probabilities))

# Length
mylogit <- glm( presenceChlam ~ length  , data = logisticDataSeabream, family =
"binomial")
summary(mylogit)
confint(mylogit)
plot(mylogit)
exp(coef(mylogit))


probabilities <- predict(mylogit, type = "response")
predicted.classes <- ifelse(probabilities > 0.5, "pos", "neg")
head(predicted.classes)
logit = log(probabilities/(1-probabilities))

# Multi-variable
mylogit <- glm( presenceChlam ~ temp + weight  , data = logisticDataSeabream, family =
"binomial")
summary(mylogit)
confint(mylogit)
plot(mylogit)
exp(coef(mylogit))


probabilities <- predict(mylogit, type = "response")
predicted.classes <- ifelse(probabilities > 0.5, "pos", "neg")
head(predicted.classes)
logit = log(probabilities/(1-probabilities))
```
